# Supplementary material for: Chromothripsis during telomere crisis is independent of NHEJ, and consistent with a replicative origin
Source: Genome Res. 2019 May;29(5):737–49. doi: 10.1101/gr.240705.118 (PMC6499312; doi:10.1101/gr.240705.118)
Supplement: Supplemental Material [file supp_gr.240705.118_Supplemental_file_1.zip › contigs/annotated_contigs/DB114/contig.2.DB114_length_585_mean_cov_11.7401709402.docx]

**DB114_length_585_mean_cov_11.7401709402**

```
ATATATCCAAAATAAAGGAAACTAGTGTGTCAAATAGATATCTGCACTCCCATGTTTATTGCAGGTCTATTCACAATAGCCAAGATATG
 >chrX:115622503-115622710 - E=7e-113
GAATCAACCTAGTGCTCATCAATGGATGAATGGATTAAAAAAGATATACACAATAGGATTTAGCCTTAAAAAGAAATAAAAGCCTATTA

TTTGCAGCAGCATGGATGAAACTAGAGGA|TACT|AGTTGGTATCCTGAAACTAAATCTAAATCCCCTTCTAAAGAAGGGTGATAGATA
                                  >chrX:115620659-115621033 - E=2e-213
TCCACAAAGATTGAGCTATATCCCCACAATACTCATTCCTTTTCTCCTTGGTAGTATACCATAAATTTTGGTTGAGCATATTGGCCACT

TAGAATAAACATAGTATTTCTTAGCTTCCTTTGCAGTGAGCTTTGGCCATGTAACTATACTCTCGCCAGTAAAATGAAAGTGGCACCAT

ACACAATGTTTGGGAAATGTACTTAAAAGGGAGGAGGCATGTCCTTCTTTACTTTTTCCGCAAGTGTCGTAGCTGAGAGCAGTTCAGCA

AACATTTTGGACAATGAAGTTGAAGATATACGTTGAGGATGAAAAAGAAACTG
```
